# Brain-Derived Neurotrophic Factor from Bone Marrow-Derived Cells Promotes Post-Injury Repair of Peripheral Nerve

**DOI:** 10.1371/journal.pone.0044592

**Published:** 2012-09-19

**Authors:** Yoshinori Takemura, Shinji Imai, Hideto Kojima, Miwako Katagi, Isamu Yamakawa, Toshiyuki Kasahara, Hiroshi Urabe, Tomoya Terashima, Hitoshi Yasuda, Lawrence Chan, Hiroshi Kimura, Yoshitaka Matsusue

**Affiliations:** 1 Department of Orthopaedic Surgery, Shiga University of Medical Science, Otsu, Shiga, Japan; 2 Department of Molecular Genetics in Medicine, Shiga University of Medical Science, Otsu, Shiga, Japan; 3 Department of Internal Medicine, Shiga University of Medical Science, Otsu, Shiga, Japan; 4 Department of Community Life Nursing, Shiga University of Medical Science, Otsu, Shiga, Japan; 5 Division of Diabetes, Endocrinology, and Metabolism, Department of Medicine, Baylor College of Medicine, Houston, Texas, United States of America; University of Medicine and Dentistry of New Jersey, United States of America

## Abstract

Brain-derived neurotrophic factor (BDNF) stimulates peripheral nerve regeneration. However, the origin of BNDF and its precise effect on nerve repair have not been clarified. In this study, we examined the role of BDNF from bone marrow-derived cells (BMDCs) in post-injury nerve repair. Control and heterozygote BDNF knockout mice (BDNF+/−) received a left sciatic nerve crush using a cerebral blood clip. Especially, for the evaluation of BDNF from BMDCs, studies with bone marrow transplantation (BMT) were performed before the injury. We evaluated nerve function using a rotarod test, sciatic function index (SFI), and motor nerve conduction velocity (MNCV) simultaneously with histological nerve analyses by immunohistochemistry before and after the nerve injury until 8 weeks. BDNF production was examined by immunohistochemistry and mRNA analyses. After the nerve crush, the controls showed severe nerve dysfunction evaluated at 1 week. However, nerve function was gradually restored and reached normal levels by 8 weeks. By immunohistochemistry, BDNF expression was very faint before injury, but was dramatically increased after injury at 1 week in the distal segment from the crush site. BDNF expression was mainly co-localized with CD45 in BMDCs, which was further confirmed by the appearance of GFP-positive cells in the BMT study. Variant analysis of BDNF mRNA also confirmed this finding. BDNF+/− mice showed a loss of function with delayed histological recovery and BDNF+/+→BDNF+/− BMT mice showed complete recovery both functionally and histologically. These results suggested that the attenuated recovery of the BDNF+/− mice was rescued by the transplantation of BMCs and that BDNF from BMDCs has an essential role in nerve repair.

## Introduction

A wide variety of studies have further characterized the repair process of peripheral nerve injury after the original study by Seddon [1]. However, a satisfactory intervention has not been developed to augment the repair of damaged peripheral nerves because the physiological processes underlying nerve regeneration, especially key molecules, have not been clarified.

Brain-derived neurotrophic factor (BDNF) [2] is a member of the neurotrophin family and has an important role in the maintenance and formation of neuronal synapses in the brain [3]. Glial cells express and respond to BDNF stimulation, favoring the notion of their pivotal role in neuroprotection [4]. BDNF helps to maintain cortical neuron size and dendrite structure rather than the initial development of these features [5]. Physiological roles for BDNF in peripheral nerves have also been suggested. BDNF enhances axonal regeneration in vitro [6] and promotes axonal sprouting from the proximal end of cut nerves into denervated nerve stumps [7]. Neurotrophic factors also affect Schwann cells in the distal nerve stump, including the promotion of Schwann cell migration [8]. Schwann cells modulate axonal sprouting by releasing BDNF, thereby promoting nerve regeneration [9]. BDNF is a crucial signaling molecule between microglia and neurons in neuropathic pain [10].

However, the main cell source and the clinical usefulness of BDNF in peripheral nerve regeneration have not been clearly demonstrated. A mouse model for BDNF deficiency has been used to evaluate the physiological roles of BDNF [11]. Homozygotic deficiency of BDNF is lethal, but heterozygotes survive. Dysregulated appetite control and obesity are reported in heterozygotes [12]. However, the relationship between BDNF deficiency and nerve regeneration following injury has not been clearly characterized. Mouse BDNF has many splice variants, and the tissue specific expression of these variants has been reported [13]. In the brain, different lesions have different expression patterns, while peripheral tissues, such as spleen, spinal cord, kidney, and liver, also express these variants in a tissue- specific manner [14].Therefore, evaluating the expression patterns of these variants in mouse regenerating nerve will provide supporting evidence of the cell source and the main players in nerve regeneration.

Recent tissue engineering data suggest that bone marrow cells (BMCs) are implicated in nerve regeneration. Direct transplantation of BMCs to the site of peripheral nerve injury augments the repair process [15]–[16]. These accumulating data led us to hypothesize that endogenous BMCs are recruited to the site of nerve injury to facilitate regeneration. Especially, BDNF production from such bone marrow derived cells (BMDCs) may have an important role in this process.

In this study, we found dramatic BDNF expression during nerve regeneration. Moreover, we observed that BMDCs were the source of BDNF by using bone marrow (BM) chimeric mice in which BMCs were selectively labeled with green fluorescent protein (GFP).

## Materials and Methods

### Animals

The procedures were approved by the Institutional Animal Care and Use Committee of Shiga University of Medical Science. Nine-week-old male C57BL/6 mice (BDNF+/+), male GFP transgenic C57BL/6 mice (BDNF+/+) (Japan SLC, Tokyo, Japan), heterozygote BDNF knockout mice (BDNF+/−) (The Jackson Laboratory, Bar Harbor, Maine), and littermate mice of BDNF+/− (BDNF+/+) were used in this study.

### Left sciatic nerve crush

**Figure 1:**
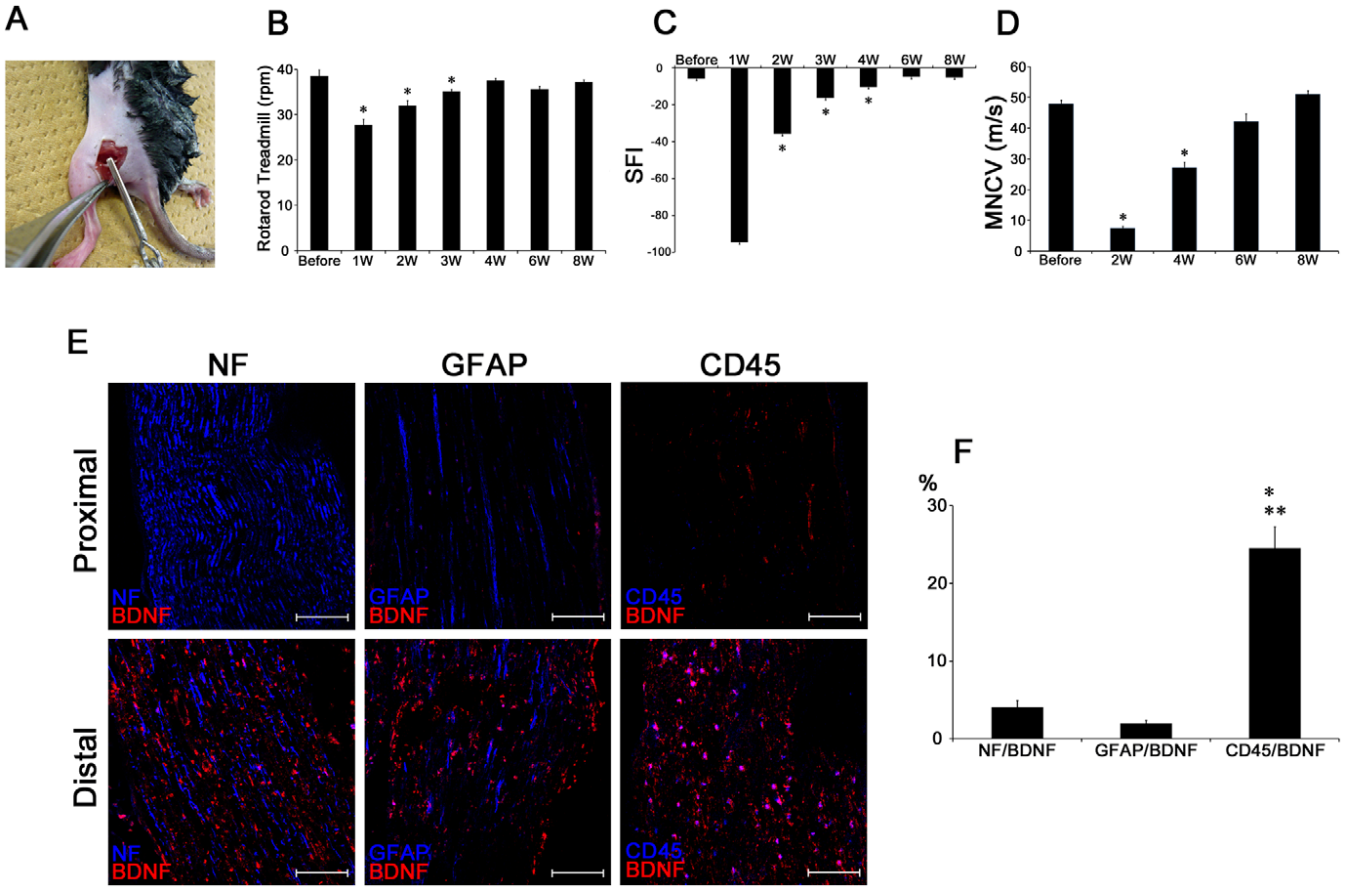
Functional and histological changes of sciatic nerve after injury. **A**) Left sciatic nerve crush using a cerebral blood clip. **B**) The maximum rpm of the rotarod treadmill test were measured for C57BL/6 mice (BDNF+/+) (n = 10). **C**) Sciatic Function Index of C57BL/6 mice (BDNF+/+) (n = 10). **D**) Motor Nerve Conduction Velocity of C57BL/6 mice (BDNF+/+) (n = 10). **E**) Overlapping staining of NF/BDNF, GFAP/BDNF and CD45/BDNF. The sections were from the sciatic nerve at 1 week after the injury. Bar indicates 100 µm. **F**) Ratio of NF-, GFAP-, CD45-, and BDNF-positive areas to the BDNF positive area. Data are the mean ± SEM. *p<0.05. ** p<0.05.

Mice were anesthetized with sodium pentobarbital (0.1 mg/100 g) intraperitoneally. The left sciatic nerve was isolated from the surrounding tissue under sterile conditions and crushed using a cerebral blood clip (MIZUHO, Tokyo, Japan) for 60 s [17]. After the operation, the skin was closed with 7-0 nylon sutures (Fig. 1a).

### Bone marrow transfer

Wild type C57BL/6 mice were irradiated (9 Gy) and then injected intravenously with 4×10^6^ BMCs isolated from male GFP mice. We called this animal model the GFP→BDNF+/+. BDNF+/− were irradiated (9 Gy) and then injected intravenously with 4×10^6^ BMCs isolated from male wild-type C57BL/6 mice (BDNF+/+). We called this animal model the BDNF+/− with bone marrow transplantation (BMT, BDNF+/+→BDNF+/−).

The nerve repair experiments were performed a month after BMT as it took about one month for the BMT to restore normal BM function in the lethally irradiate mice [18].

### Immunohistochemistry

The control and GFP→BDNF+/+ mice were deeply anaesthetized. The left sciatic nerves were collected at 1, 2, 3, 4, 6 and 8 weeks after injury. The tissue samples were washed in phosphate-buffered saline (PBS) three times, embedded in OCT compound (Tissue Tek, Sakura Finetek USA), sectioned at 7 µm thickness, and prepared for staining. For overlapping immunofluorescence, we incubated frozen sections with antibodies against BDNF (rabbit polyclonal, Abcam, Ltd., Cambridge, U.K. and Santa Cruz Biotechnology, INC., Delaware, CA, USA ) and CD45 (rat and mouse monoclonal, BD Biosciences, San Diego, USA), neurofilament (NF) (rabbit polyclonal, Abcam, Ltd., Cambridge, U.K.), or GFAP (rabbit polyclonal, Abcam, Ltd., Cambridge, U.K.). The sections were then incubated with fluorescence-labeled secondary antibodies and photomicrographs were obtained from the specimens under a confocal laser-scanning microscope (LSM510, Leica, Germany). Negative controls included omission of primary or secondary antibodies on parallel sections.

### Morphologic and morphometric analyses

The mice were deeply anaesthetized. The left sciatic nerves were collected at 4, 6 and 8 weeks after injury. Four mm segments that were proximal and distal to the lesion site were dissected, washed three times in PBS and then further fixed in 2% osmium tetroxide for 30 min. The segments were prestained with uranyl acetate for 3 h, dehydrated in serial acetone solutions and embedded in Epon resin. Semi-thin (1 µm) transverse sections were cut from the segment 4 mm distal to the lesion site using an ultramicrotome, and stained with toluidine blue. Images of the distal portion of the crush site were captured and digitized using a microscope (Eclipse E-600; Nikon, Japan) coupled to a high-performance CCD camera and processed with Image-Pro Plus software, version 6.0 (Media Cybernetics, Bethesda, MD, USA). Morphometric measurements of the sciatic nerve included: (1) average myelinated fiber area (µm^2^); and (2) average counts of myelinated fibers (/100 µm^2^). These morphological parameters were chosen to assess the differentiation of the regenerating sciatic nerves.

### Sciatic function Index

Analysis of the walking pattern of a mouse by recording its footprints and calculating the Sciatic Function Index (SFI) is a well-established and commonly used method to assess motor nerve recovery after sciatic nerve injury [19]. To obtain the walking pattern, the hind paws were pressed onto an inkpad (Shachihata, Tokyo, Japan), and the mice were allowed to walk up a small inclining gang way (slope 20°, length 50 cm, width 14 cm), which was lined with white paper (modified from [20]). All mice had a few pre-experiment training runs. The walking track experiment was carried out before, and 1, 2, 3, 4, 6, and 8 weeks after the sciatic nerve crush. The following footprint parameters were measured by using a ruler: (1) print length (PL, distance from the heel to the third toe), (2) toe spread (TS, distance from the first to the fifth toe), and (3) intermediate toe spread (ITS, distance from the second to the fourth toe). All these measurements were taken from the left experimental paw (EPL, ETS and EITS, respectively) and from the right non- operated paw (NPL, NTS and NITS, respectively) of each mouse. By using these data, the SFI, which reflects the differences between the injured and the intact contralateral paw, was calculated by the modified formula [21].
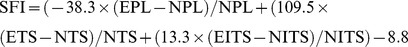
An SFI of nearly 0 is normal, while an SFI of −100 indicates total impairment of the sciatic nerve.

### Rotarod treadmill test

Motor coordination and balance were tested on a rotarod apparatus (Ugo Basile, Collegeville, PA, Italy). Performance was tested before, and 1, 2, 3, 4, 6, and 8 weeks after the sciatic nerve crush. The rod (diameter, 7.0 cm) was accelerated at 0.15 rpm and increased in speed from 4 to 40 rpm at a constant rate of acceleration over 4 min [22]. Scores (maximum rpm achieved) were averaged over 7 trials per session [23].

### Motor nerve conduction study (MNCS)

For an electro-diagnostic examination, a portable electro-diagnostic device was used (NIHON KODEN, Tokyo, Japan). The animals were anesthetized for the exposure of the lesioned sciatic nerve. Body temperature was maintained by keeping the mice on an electric device. The ground electrode was placed subcutaneously in the stomach. The two recording electrodes were inserted in the Achilles tendon and the belly of the gastrocnemius muscle. The exact placement of the recording and stimulation electrodes is important, because co-stimulation of the proximal muscle branch of the sciatic nerve or co-recording from neighboring muscles could result in false positive signals. For proximal stimulation, the two stimulation electrodes were placed close to the sciatic nerve near the hip joint between the major trochanter and ischial tuberosity. For distal stimulation, the electrodes were placed close to the nerve near the popliteal fossa. The electro diagnostic device automatically calculated the CMAP amplitude and the motor nerve conduction velocity (MNCV) from the latency difference and the distance (in mm) between the proximal and distal stimulation positions. The MNCV, CMAP amplitudes and distal latencies were was calculated before, and 1, 2, 4, 6 and 8, weeks after the sciatic nerve crush.

### RT-PCR

The C57BL/6 (BDNF+/+) mice were sacrificed and the left sciatic nerves distal to the crush site were extracted immediately at 1, 2, 3, 4, 6, and 8 weeks after injury. The BMCs and the intact sciatic nerve were taken from the left femur of control C57BL/6 mice. RNA was extracted from the distal portion of the crush site and BMCs, and reverse transcribed using SuperScript 3™ (Invitrogen, INC. Carlsbad, CA, USA) reverse transcriptase, according to the manufacturer's instructions. First-strand complementary RNA was synthesized by using 0.5 µg oligo (dT) primers, 10× first-strand buffer, 0.01 M dithiothreitol, 0.5 mM deoxyribonucleotide mix, and 200 U of SuperScript 3™ at 50°C for 50 min. The reaction was stopped by heating at 85°C for 5 min. Each sample was amplified by performing 35 cycles of the polymerase chain reaction (PCR) using the same oligonucleotide primers as previously reported [13]. The amplification conditions were as follows: initial denaturation was performed at 94°C for 5 min, followed by 35 cycles of denaturation (at 94°C at 30 s), annealing (at 56°C for BDNF splice variants 6A and 6B/at 64°C for BDNF splice variants 1, 2A, 2B, 2C, 3, 4, and 5) for 30 s, extension (at 72°C for 30 s), and final elongation (at 72°C for 7 min). The amplified products were resolved on an agarose gel (1.5%) by performing homeothermic gel electrophoresis at 100 V for 30 min.

### QT-PCR

Quantitative-PCR was performed on the LightCycler System (Roche Diagnostics, Basel, Switzerland) using a LightCycler 480 Probes Master kit (Roche Diagnostics, Basel, Switzerland) following the manufacturer's protocol. The reaction was performed in a 20 µl mixture containing 1 µl of the above-synthesized cDNA and 19 µl master mix. For BDNF (splice variant 5), the cDNA sample was amplified using specific primers (Variant 5 Taqman Forward: CAGAAGCGTGACAACAATGTGA, Reverse: ACCATAGTAAGGAAAAGGATGGTCAT, Probe: ACCCTGAGTTCCACCAGG). After an initial denaturation step at 95°C for 10 min, amplification was performed using 50 cycles of denaturation (95°C for 10 s), annealing (64 °C for 15 s), and extension (72 °C for 10 s).

### Statistical analysis

The Results are expressed as mean values±standard error (SE). One-way analysis of variance with a Bonferroni/Dunn post hoc test was used to determine significant differences among the groups. A *P-value* less than 0.05 was considered to be significant.

## Results

### Relationship between nerve functional recovery and BDNF expression after injury

**Figure 2:**
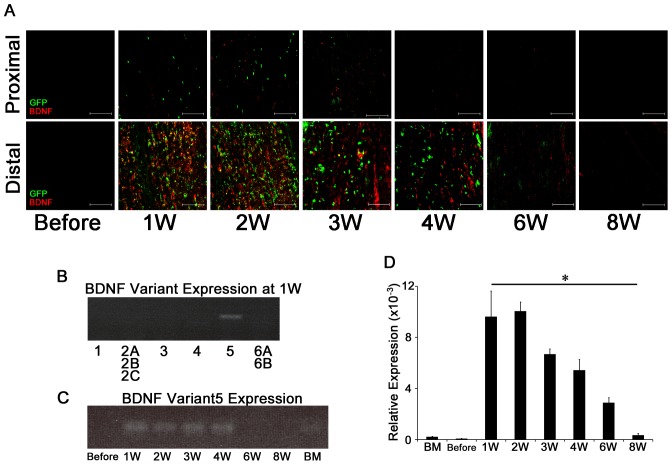
Effect of BMDCs in sciatic nerve recovery from injury. **A**) GFP and BDNF immunohistochemistry in the C57BL/6 mice (BDNF+/+). Bar indicates 100 µm. **B**) The expression of the BDNF splice variants at 1 week. **C**) The expression of BDNF variant 5 throughout the experimental period. **D**) Relative expression level of BDNF variant 5 in the sciatic nerve from C57BL/6 mice (BDNF+/+). (n = 10). Data are the mean ± SEM. * p<0.05.

The left sciatic nerve was isolated and crushed using a cerebral blood clip for 60 s in normal C57BL/6 mice (Fig. 1A). Sequential changes of sciatic nerve function were evaluated using 3 functional tests; rotarod treadmill test (Fig. 1B), SFI (Fig. 1C) and MNCV (Fig. 1D). First, motor coordination and balance were tested on a rotarod apparatus. Before sciatic nerve crush, all mice could stay on the rod until approximately 40 rpm (240 s). The parameter was expressed as the maximum endurable rpm, at which the animals fell off the rotating rod. After the nerve injury, the mice suddenly showed a clear impairment of nerve function (Fig. 1B). The endurable rpm was significantly decreased by 27% at 1 week after the sciatic nerve crush; however, the mice showed signs of recovery at 2 weeks, and they achieved full recovery at 4 weeks. Second, at 1 week after the sciatic nerve crush, the average SFI decreased to as low as −90 points, where an SFI of −100 points represents complete paralysis (Fig. 1C). The signs of motor function recovery could be seen as early as 2 weeks after the injury, where the SFI returned to −40 points. After 2 weeks, the animals demonstrated a gradual return of motor function over time and achieved almost full recovery at 6 weeks, suggesting that the nerve injury in our study represented axonotmesis. After 8 weeks, the SFI score had recovered to the normal level. Third, a nerve conduction study is an established method to quantify peripheral nerve dysfunction. After the sciatic nerve crush, the MNCV of the mice became immeasurable at 1week. The MNCV then became detectable at 2 weeks, and gradually increased thereafter (Fig. 2D). After 6 weeks, the MNCV reached the same level of the intact nerve.

Next, we examined the injured nerve by histological analysis to evaluate BDNF expression at 1week in the proximal and distal sites of the nerve injury. In addition, we performed double staining for BDNF and NF for axons, BDNF and GFAP for Schwann cells, and BDNF and CD45 for BMDCs (Fig. 1E). BDNF was mainly expressed in the distal segment from the crush site (Fig. 1E). After the division of the overlapped area by the total BDNF-positive area for each cell type, we evaluated the origin of BDNF production. Interestingly, BDNF was mainly expressed in the distal segment of the nerve tissue (Fig. 1f), and CD45 positive cells were the main source of this BDNF. We confirmed almost same result on using BDNF antibody supplied from 2 companies (Abcam and Santa Cruz).

### Expression of BDNF during Sciatic Nerve Repair in GFP→BDNF+/+ mice

Since CD45-positive cells were thought to be the main source of BDNF in the early recovery phase after sciatic nerve injury, we examined its expression in BMDCs before and at 1, 2, 3, 4, 6, and 8 weeks after nerve injury in GFP→BDNF+/+ mice, in which BMCs were selectively labeled with GFP. As shown in Fig. 2A, there were very few BMDCs and BDNF-positive cells in the proximal segment compared with those in the distal segment. Furthermore, in the sciatic nerve before the crush, we hardly observed BDNF and GFP expression (Fig. 2A). In contrast, striking BDNF expression was clearly demonstrated after the sciatic nerve injury at the distal segment from the crush site. Following the sequential changes of BDNF expression after the nerve crush, the peak in its expression was observed at 1–2 weeks (Fig. 2A). The intact sciatic nerve was devoid of GFP-positive cells, suggesting that the BMDCs do not normally appear in the sciatic nerve. Furthermore, since the sequential change of GFP expression clearly overlapped with that of BDNF expression and its peak was observed at 1–2 weeks (Fig. 2A), we divided number of double positive cells (i.e., BDNF and GFP) by the number of total GFP-positive cells during the course of the recovery; before: 0%, 1 week: 31%, 2 weeks: 35%, 3 weeks: 21%, 4 weeks: 12%, 6 weeks: <10%, and 8 weeks: 0%. These results indicate that BDNF production by BMDCs may play a crucial role in the recovery from the nerve injury.

To further support our hypothesis that the BMDCs are the main source of BDNF expression during nerve regeneration, we examined the expression profiles of tissue-specific BDNF mRNA splice variants at 1 week. By RT-PCR analysis for BDNF mRNA, only variant 5 was expressed in the sciatic nerve tissue (Fig. 2B). The expression of variant 5 became evident in the injured nerve from 1–4 weeks after the nerve crush (Fig. 2C). No other variants were detected during nerve regeneration (data not shown). We then determined the sequential changes in the mRNA expression of variant 5 by QT-PCR, and the result clearly confirmed the RT-PCR expression pattern (Fig. 2D). The peak expression of variant 5 at 1–2 weeks clearly coincided with the prominent accumulation of GFP-positive cells that were recruited from the BM.

### Functional recovery of the peripheral nerve in BDNF+/− after the nerve injury

Our data indicated that BDNF expression from BMDCs may play an important role in nerve recovery after injury. Therefore, we then studied the “loss of function” of the BDNF+/− mice and examined the effect of BMT from BDNF+/+ mice after peripheral nerve injury. In this study, we performed the same tests as were administered to the control mice (i.e., rotarod treadmill test, SFI, andMNCS) among 3 groups of mice: BDNF-deficient heterozygotes (BDNF+/−), their littermates (BDNF+/+) and BDNF-deficient heterozygotes with BMT from normal mice (BDNF+/+→BDNF+/− mice).

**Figure 3:**
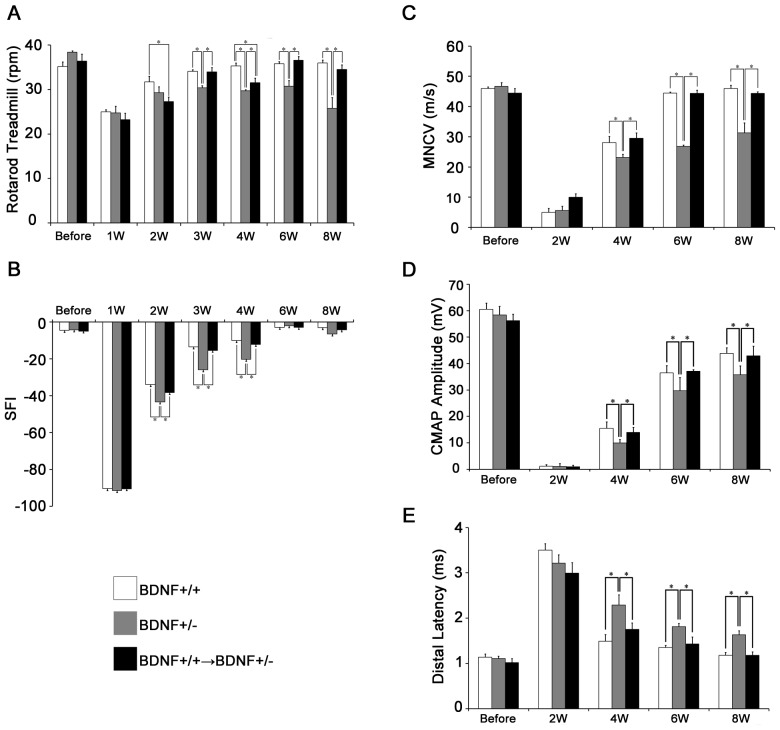
Functional changes of sciatic nerve in BDNF+/−. **A**) The maximum rpm of the rotarod treadmill test were measured for littermates (BDNF+/+), BDNF+/− and BDNF+/+→BDNF+/− (n = 5 per genotype). **B**) The SFI of the same groups of mice. (n = 5 per each group). **C**) Motor Nerve Conduction Velocity of the same groups of mice. (n = 5 per each group). **D**) CMAP amplitude of the same groups of mice (n = 5 per each group). **E**) Distal latency of the same groups of mice (n = 5 per each group). Data are the mean ± SEM. *p<0.05.

In the rotarod treadmill test, there were no significant differences among the 3 groups (Fig. 3A). The endurable rpm was clearly decreased after the sciatic nerve crush in all experimental groups, suggesting the consistency of the crush injury. All mice showed the first signs of recovery in the rotarod treadmill test at 2 weeks. The littermates achieved full recovery at 4 weeks as observed in the C57BL/6 mice (BDNF+/+) (Fig. 1B). In contrast, the BDNF+/− fell off the rod significantly faster than their littermates at 3 weeks, and failed to achieve full recovery at 8 weeks. However, from 3 weeks on, the BDNF+/+→BDNF+/− could stay on the rod significantly longer than the BDNF+/−, and achieved full recovery at 6 weeks, similar to their littermates (BDNF+/+). These results suggested that the impaired recovery from the nerve injury for motor coordination and balance in the absence of BDNF was rescued by the transplantation of BMCs.

Without injury, the BDNF+/− mice demonstrated a normal gait in the walking alley, and their SFI was not altered as compared with the other experimental groups (Fig. 3B). At 1 week after the injury, all experimental groups demonstrated prominent decreases in SFI, suggesting the consistency of the crush injury. After 2 weeks, all animals demonstrated a gradual return of motor function over time and achieved almost full recovery at 6 weeks. However, the BDNF+/− mice showed significantly lower SFI scores than the other two groups at 2–4 weeks. This result indicates that the motor function recovery of BDNF+/− mice is delayed compared with their littermates. However, at 3 and 4 weeks, the BDNF+/+→BDNF+/− mice showed improved SFI scores and no significant differences compared with their littermates.

No significant differences in the MNCV, CMAP amplitude and distal latency before sciatic nerve crush were found among the 3 groups (Fig. 3C, 3D and 3E). At 1week after the crush, the sciatic nerve of the BDNF+/+→BDNF+/− mice was not electrically excitable similarly to the other groups. The MNCV of all mice became detectable at 2 weeks, and gradually increased until 8 weeks. After 6 weeks, the MNCV of the littermates reached the level of the intact nerve. In contrast to the linear recovery of the MNCV in the littermates, the recovery of MNCV in the BDNF+/− mice was reduced at 4 weeks. Furthermore, at 6 and 8 weeks, the MNCV of the BDNF+/− mice was still significantly lower than the littermates, suggesting retarded nerve regeneration in the BDNF+/− mice as compared with their littermates. On the other hand, the MNCV of the BDNF+/+→BDNF+/− mice gradually increased and achieved full recovery at 6 weeks. These changes of the MNCV were similar to those of the littermates (BDNF+/+). There were no significant differences in the MNCV between the littermates and BDNF+/+→BDNF+/− mice throughout the experimental period.

The CMAP of all mice became detectable at 2 weeks, with gradually increasing amplitude until 8 weeks, but did not achieve full recovery at 8 weeks. We note that the CMAP amplitude of BDNF+/− mice was significantly lower than that of BDNF+/+ and BDNF+/+→BDNF+/− mice from 4 weeks to 8 weeks (Fig. 3D).

The distal latency of all mice became detectable at 2 weeks, and gradually increased until 8 weeks, but did not achieve full recovery at 8 weeks. The distal latencies of BDNF+/− mice were significantly longer than that of BDNF+/+ and BDNF+/+→BDNF+/− mice from 4 weeks to 8 weeks (Fig. 3E).

These results suggested that the attenuated recovery of the nerve conduction study in the BDNF+/− mice was rescued by the transplantation of BMCs.

### Morphological recovery of the peripheral nerve in BDNF+/− mice after nerve injury

**Figure 4:**
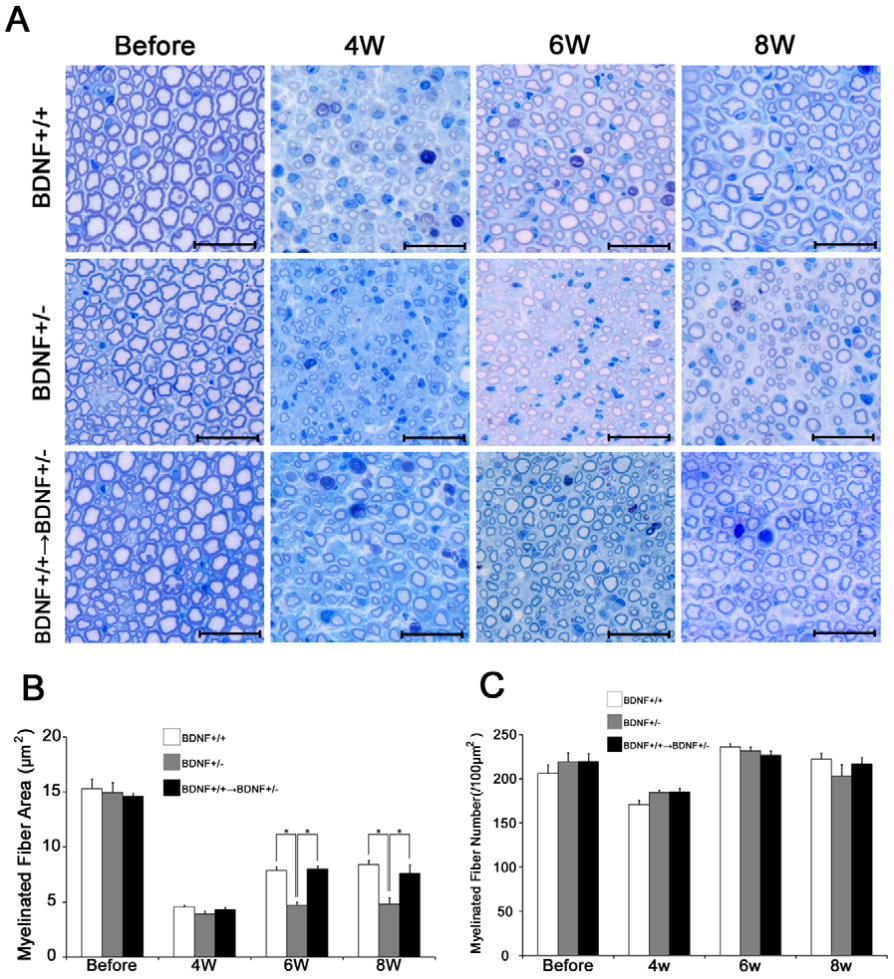
Histological changes of sciatic nerve in BDNF+/−. **A**) Nerve fiber regeneration was examined in BDNF+/+ littermates, BDNF+/−, and BDNF+/+→BDNF+/−. (n = 5 per genotype). Bar indicates 20 µm. **B**) Myelinated fiber area in each group. (n = 5 per each groups). **C**) Number of myelinated fibers in each group. (n = 5 per each groups). **p<0.05.

Before the sciatic nerve crush, there were no significant differences in the area and number of myelinated fibers among the 3 groups of mice, i.e., BDNF+/−, BDNF+/+ littermates, and BDNF+/+→BDNF+/− mice, suggesting that the BDNF-deficient mice have no inherent histological abnormalities (Fig. 4A–4C). After the nerve crush injury, regenerated nerve fibers were not detected distal to the crush site (data not shown) in all groups at 2 weeks. However, regenerated myelinated fibers could be detected at 4 weeks (Fig. 4A). The area of myelinated fibers decreased at 4 weeks and there was no significant difference among the 3 groups (Fig. 4A and 4B). After 6 weeks, by histology the area of myelinated fibers of the littermates increased, although it did not reach the same level as the sciatic nerve before the injury (Fig. 4A and 4B). In contrast to the clear increase of the area of myelinated fibers of the BDNF+/+ littermates, the BDNF+/− mice showed no increase after 6 weeks (Fig. 4A and 4B). At 6 or 8 weeks, the average area of myelinated fibers of the BDNF+/− mice was smaller than that of the littermates, suggesting attenuated nerve regeneration in the BDNF+/− mice. In contrast, the average area of myelinated fibers of the BDNF+/+→BDNF+/− mice increased and reached the same level as the littermates (Fig. 4A and 4B), suggesting that attenuated regeneration of myelinated nerves in the BDNF+/− was rescued by BMT.

The average number of myelinated fibers of the 3 groups showed no significant differences before the sciatic nerve crush (Fig. 4A and 4C). At 2 weeks, we did not detect myelinated fibers distal to the crush site (data not shown). At 4 weeks, regenerated myelinated fibers were detected in all groups, and the average number of fibers was decreased in all groups as compared with the mice before the injury. After 6 weeks, the average number of myelinated fibers of all groups reached the same level as observed before the crush. There were no significant differences between the number of myelinated fibers among the groups throughout the experimental period (Fig. 4C).

## Discussion

We investigated the recruitment of BMDCs to the site of a peripheral nerve injury and the expression of BDNF during nerve regeneration, and found that the expression of BDNF coincided spatiotemporally with the accumulation of recruited BMCs by using GFP→BDNF+/+ mice. Furthermore, we characterized the expression of BDNF splice variants at the site of nerve regeneration, and found that the specific variant expressed at the site of nerve regeneration is also specifically expressed in the bone marrow. These observations further led us to hypothesize that endogenous BMCs are implicated in nerve regeneration *via* the expression of BDNF. We then studied the repair process of peripheral nerve injury using BDNF+/− mice. The impaired nerve repair observed in the BDNF+/− mice was consistent with an attenuated function of BDNF in these mice. In contrast, BDNF+/+→BDNF+/− mice showed complete functional and histological recovery. These observations strongly supported the view that BDNF from BMDCs plays a pivotal role in the repair of peripheral nerve injury.

Previous studies have demonstrated that BDNF protein secreted from neurons in the dorsal root ganglia [24]–[25] and the anterior horn of the spinal cord [26] was transported to the regeneration site *via* axons. It has also been demonstrated that BDNF protein secreted at the neuromuscular junction was transported *via* axons to the regeneration site [27]. It is thus possible that the BDNF protein identified at the crush site in the present study might have been transported there in an antegrade and/or retrograde manner. However, in this study, BDNF expression at the nerve regeneration site was largely co-localized with CD45, a common marker of BMDCs, and with GFP-positive cells in the BMT study. Furthermore, we studied the expression of BDNF mRNA and its splice variants in the regenerating nerve. Murine BDNF mRNA consists of 9 different splice variants, and all of these variants have been detected in brain tissue [13]. In the present study, we also detected all BDNF splice variants in the brain tissue of healthy animals (data not shown). According to our review of the literature, there have been no reports studying the expression of BDNF splice variants during peripheral nerve regeneration. We detected the expression of only BDNF splice variant 5 in the regenerating nerve. Most interestingly, BDNF splice variant 5 was also the only variant detected in the BMCs. Since our confocal microscopy study demonstrated that the accumulation of BDNF in the injured nerve colocalized with the GFP-positive cells, BDNF splice variant 5 is likely to be derived from the BMDCs. These results indicate that the main source of BDNF during peripheral nerve regeneration is BMDCs.

In order to characterize the function of BDNF during peripheral nerve regeneration, it is very important to analyze the relationship between the functional recovery and histological changes. Although the delay of nerve regeneration was reported recently using BDNF+/− mice, as we did in our study, the functional and histological relationship was not clearly demonstrated. Here we investigated the precise repair process using BDNF+/− mice, and found that the mice showed significantly lower scores for the SFI, rotarod treadmill test, and MNCS than their BDNF+/+ littermates. These abnormalities in BDNF+/− were completely restored by BMT; however, the BDNF+/− mice only showed complete recovery of the SFI after 6 weeks. This finding might be related to the limited sensitivity of the SFI. As for the rotarod treadmill test, similar results were obtained at 2–4 weeks. Therefore, we employed these traditional methods for functional evaluation in conjunction with MNCS and histomorphometric analysis for the overall assessment of this model.

The area of myelinated fibers was decreased at 4 weeks and there was no significant difference between the 3 groups (Fig. 4A and 4B). After 6 weeks, the area of myelinated fibers of the BDNF+/+ littermates increased, although it did not reach the level observed before the injury (Fig. 4A and 4B). In contrast, the area of myelinated fibers of BDNF+/− mice showed no increase after 6 weeks (Fig. 4A and 4B). There was no significant difference in the average number of myelinated fibers between the 3 groups throughout the experimental period, indicating that the deficiency of BDNF of BDNF+/− mice is not related to the recovery in the number of the myelinated fibers. These results indicated that BDNF from BMDCs is necessary for nerve regeneration.

For the experimental treatment of peripheral nerve palsy, replacement therapy using progenitor cells, such as the transfer of BMCs [16], mesenchymal stem cells [28] or adipose-derived stem cells [29] to the injured peripheral nerve site, or intrathecal injection of BMCs to the spinal cord have been conducted, demonstrating variable degrees of functional improvement [30]. Recently, the effect of cell therapy on nerve regeneration using BDNF gene-transferred neural stem cells was reported [31]. Furthermore, activity-dependent therapy using electrostimulation or exercise reportedly stimulates axonal growth through the endogenous secretion of BDNF. These newly developed methods might open up a therapeutic paradigm for cell therapy of peripheral nerve injuries.
